# Novel Mechanisms of SARS-CoV-2 Drug Resistance and Rational Design of Anti-Resistant Antivirals

**DOI:** 10.3390/molecules31152655

**Published:** 2026-07-30

**Authors:** Xianghan Bai, Bing Ye, Shenghua Gao, Peng Zhan, Xinyong Liu

**Affiliations:** State Key Laboratory of Discovery and Utilization of Functional Components in Traditional Chinese Medicine, Key Laboratory of Chemical Biology (Ministry of Education), Department of Medicinal Chemistry, School of Pharmaceutical Sciences, Shandong University, Ji’nan 250012, China; 202416771@sdu.edu.cn (X.B.); yebing1106@163.com (B.Y.)

**Keywords:** SARS-CoV-2, drug resistance, main protease, exoribonuclease, nucleoside analogues, structure-based drug design

## Abstract

Antiviral drug resistance in SARS-CoV-2 is increasingly limiting treatment efficacy. Four recent studies have revealed two key resistance mechanisms: (1) Mutations in the main protease (M^pro^)—including E166V, E166A, and S144-series variants—disrupt drug binding or active-site conformation, reducing nirmatrelvir efficacy. (2) The proofreading exoribonuclease (ExoN) removes incorporated nucleoside analogues (e.g., bemnifosbuvir, sofosbuvir), conferring resistance. Guided by structural and pharmacological insights, three effective countermeasures have been established: structure-based optimization of M^pro^ inhibitors, rational design of ExoN-evading nucleoside analogues, and synergistic combination therapies. These advances provide a solid framework for developing next-generation antivirals to combat emerging resistant SARS-CoV-2 variants.

Since the World Health Organization (WHO) declared COVID-19 a global pandemic in March 2020, SARS-CoV-2 has caused widespread morbidity and mortality worldwide, posing an unprecedented challenge to public health systems [1]. As a single-stranded positive-sense RNA virus, SARS-CoV-2 encodes key enzymes including main protease (M^pro^) and RNA-dependent RNA polymerase (RdRp) that serve as core targets for antiviral drug development [2,3,4]. Among clinically approved agents, the M^pro^ inhibitor Paxlovid (nirmatrelvir co-administered with ritonavir) and RdRp inhibitor remdesivir have emerged as key therapeutic options for COVID-19 [5,6,7]. However, the high mutation rate of the SARS-CoV-2 genome, combined with selective pressure from antiviral drugs and natural viral evolution, has driven the continuous emergence of drug-resistant variants, leading to reduced efficacy or even complete treatment failure. This situation underscores an urgent need to elucidate the underlying resistance mechanisms and to develop next-generation antivirals capable of overcoming resistance. Four recent studies have characterized two prevalent mechanisms of SARS-CoV-2 drug resistance: resistance to M^pro^ inhibitors mediated by key mutations in the protease, and resistance to NA antivirals conferred by the proofreading exoribonuclease (ExoN) [8,9,10]. Based on these findings, antiviral agents with strong anti-resistance potency were developed as countermeasures towards the significant efficacy reduction of approved SARS-CoV-2 inhibitors caused by viral mutations.

## 1. Drug Resistance Mechanisms Mediated by Key M^pro^ Mutations and Structure-Based Design of Anti-Resistance Agents

As a key enzyme in the SARS-CoV-2 replication cycle, M^pro^ generates functional viral proteins by cleaving the polyproteins pp1a and pp1ab. The conserved residues within its active site pocket constitute the core binding interface for inhibitor engagement [4]. Two recent studies from the Wang laboratory systematically profiled the resistance landscape of clinically relevant SARS-CoV-2 M^pro^ mutants against approved and investigational inhibitors [8,9]. In the first study, researchers evaluated ibuzatrelvir, ensitrelvir and nirmatrelvir against a panel of naturally occurring nirmatrelvir-resistant M^pro^ variants using FRET-based inhibition assays, thermal shift binding and antiviral plaque assays. The Omicron signature mutation P132H remained fully sensitive to all three inhibitors. Mutations at residues S144, Q192 and H172 conferred moderate (10- to 100-fold) resistance, whereas substitutions at E166, particularly E166A and E166V, led to strong cross-resistance. The highest resistance levels were observed in the E166A/L167F double mutant and the L50F/E166A/L167F triple mutant [9]. In the subsequent study, researchers confirmed that E166V also drives cross-resistance to multiple next-generation M^pro^ inhibitors which share a pyrrolidone or piperidone P1 moiety, including TKB-245, ML2006a4, EDP-235 and simnotrelvir. Importantly, both E166A and E166V retain enzymatic activity comparable to wild-type M^pro^, making them clinically relevant escape variants [8]. Together, these findings establish E166 mutations as a critical vulnerability of current M^pro^ inhibitors and highlight the urgent need for anti-resistance antivirals that do not depend on hydrogen bonding with this residue.

**Figure 1:**
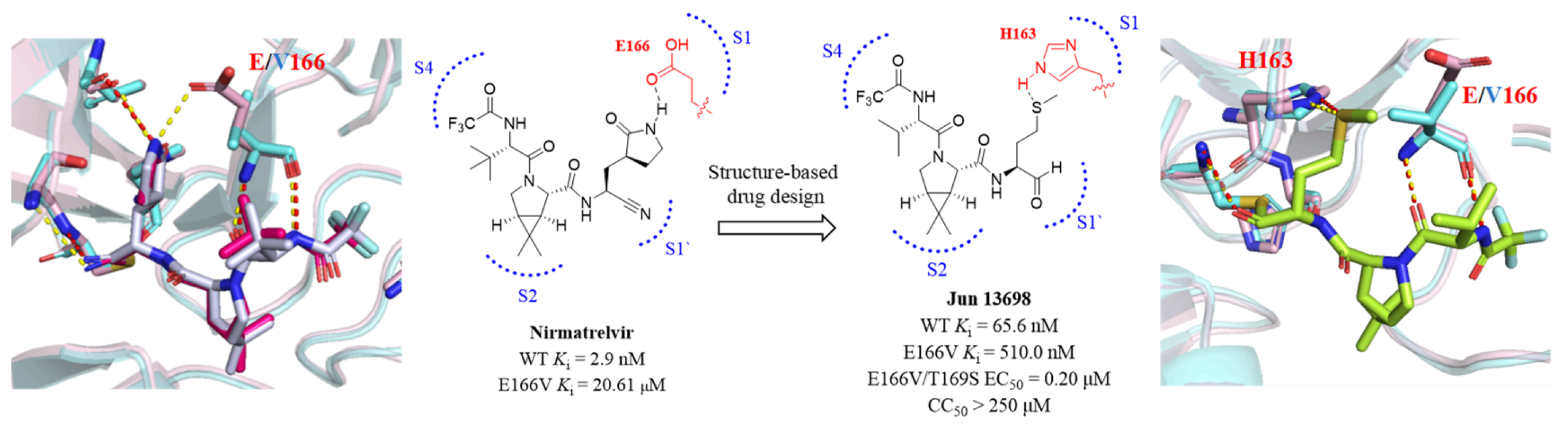
Binding modes of nirmatrelvir and Jun13698 to SARS-CoV-2 M^pro^. Superimposition of wild-type M^pro^–nirmatrelvir (PDB: 8DZ2, light pink/hot pink; yellow H-bonds) and E166V M^pro^–nirmatrelvir (PDB: 8H82, aquamarine/grey; red H-bonds). Binding pose of Jun13698 to M^pro^ (PDB: 9XYM, light pink/green; yellow H-bonds) superimposed onto E166V M^pro^ (PDB: 9XYZ, aquamarine; red H-bonds).

Building on a comprehensive understanding of resistance mechanisms and the structural features of M^pro^ mutants, the Wang research group has developed next-generation covalent M^pro^ inhibitors guided by a design strategy that bypasses critical hydrogen-bond dependencies while enhancing hydrophobic interactions [8]. Taking the optimal compound Jun13698 as an example, its key optimization lies in replacing the pyrrolidinone moiety of nirmatrelvir with a methionine side chain. This modification not only circumvents dependence on hydrogen bonding with E166 but also enables stable binding to resistant mutants such as E166V and E166A through hydrophobic contacts. X-ray crystallography and molecular dynamics simulations confirmed that Jun13698 forms multiple stabilizing interactions with the S1, S2 and S4 subsites of M^pro^: the P1 methionine side chain inserts into the S1 pocket and forms hydrophobic interactions with V166; the P2 dimethylcyclopropylproline moiety optimally complements the hydrophobic S2 pocket; and the N-terminal trifluoroacetamide group extends into the S4 pocket, hydrogen-bonding with Q192 (Figure 1). Enzymatic and cell-based assays confirmed that Jun13698 potently inhibits wild-type M^pro^ and drug-resistant mutants (e.g., S144A/M, M165T, H172Q/Y), achieving K_i_ values of 50.2–402.9 nM. Regarding recombinant viruses carrying E166V or E166A, Jun13698 also retained potent antiviral efficacy (EC_50_ = 0.20–1.01 μM) with resistance fold changes of only 2.5–3.4, substantially lower than that of nirmatrelvir (212.7-fold), and exhibited no significant cytotoxicity (CC_50_ > 250 μM) [8].

## 2. ExoN-Mediated Viral Drug Resistance and Optimization of Nucleoside Analogue Antivirals

Parallel studies by Yang’s group revealed a second core resistance mechanism: ExoN-mediated excision of nucleoside analogues (NAs). NA drugs exert their antiviral effects by acting as substrates of the RdRp; upon incorporation into the viral RNA chain, they terminate replication. However, the SARS-CoV-2 ExoN, a complex formed by nsp14 and nsp10, can excise incorporated NAs through its proofreading activity, representing a critical determinant of resistance to this class of antivirals [10,11]. Structural and biochemical analyses revealed that NA incorporation reshapes RNA binding dynamics: on the one hand, it weakens the interaction between RNA and RdRp; on the other hand, it enhances the affinity of RNA for ExoN, thereby facilitating the transfer of the RNA substrate from RdRp to ExoN and its subsequent excision [10,11]. The P140-L149 allosteric regulatory loop of ExoN serves as a key structural element governing its catalytic activity, contributing to differential levels of drug resistance [10]. Ribose ring modifications of NAs, such as the 2′-CH_3_ group, engage in hydrophobic interactions with residues P141 and F146 within this loop. These interactions disrupt the stable association between the loop and the α4-α5 helical region, ultimately leading to attenuated ExoN catalytic activity. Conversely, ExoN engages in a sequence-specific recognition of NAs through conserved structural motifs. For instance, the 1′-cyano group of remdesivir forms critical hydrogen bonds with residues H95 and N104 of ExoN, which facilitates its excision [11]. This mechanistic insight helps explain why certain NA inhibitors with potent activity against HCV, such as sofosbuvir, exhibit limited efficacy against SARS-CoV-2 [12].

**Figure 2:**
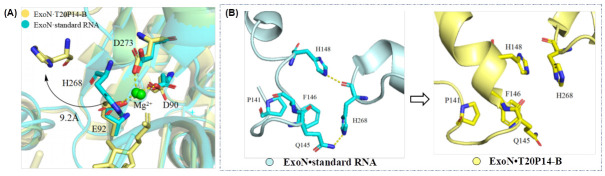
(**A**) Overlap of ExoN active site in the presence of T20P14-B (PDB:9YRK) or a standard RNA (PDB:7N0C). Root-mean-square deviation (RMSD) of the superimposition is indicated. A pronounced conformational shift of H268 upon binding of the T20P14-B ligand is observed. Cyan representations correspond to ExoN bound with standard RNA, and yellow representations represent ExoN in complex with T20P14-B. (**B**) Incorporation of bemnifosbuvir disrupts the activated conformation of ExoN, thereby conferring moderate resistance to ExoN-mediated excision.

To counter ExoN-mediated resistance to NA antivirals, optimization strategies have primarily focused on enhancing drug resilience to ExoN-catalyzed excision. Leveraging structural insights into ExoN, recent studies have demonstrated that introducing modifications capable of disrupting the activation of the P140-L149 regulatory loop can effectively impair the assembly of ExoN’s catalytic machinery [10]. Concurrently, the use of a guanine-based nucleobase scaffold has been shown to reduce recognition efficiency by ExoN, as the binding affinity of ExoN for guanine-derived NAs is significantly lower than that for uracil- or cytosine-based counterparts [10]. As an optimized NA, bemnifosbuvir bears a 2′-fluoro-2′-C-methyl modification on a ribose ring. T20P14-B, formed by hairpin T20P14 RNA incorporated with bemnifosbuvir, was found to induce a conformational shift in the P140-L149 regulatory loop of ExoN (Figure 2A) [10,13]. This shift widens the distance between the loop and the α4-α5 helix that harbours the catalytic residue H268, disrupting two critical hydrogen bonds normally formed between Q145/H148 and H268 (Figure 2B). As a result, H268 is locked in an inactivated conformation, impairing ExoN’s proofreading function and conferring moderate resistance to ExoN- mediated excision, as confirmed by cryo-EM structural studies.

## 3. Building a High Genetic Barrier to Antiviral Resistance Through Combination Therapy Strategies

Combination therapy is a well-established strategy to combat antiviral resistance. Monotherapy imposes strong selective pressure, quickly enriching resistant mutants. A thoughtfully designed combination, whether targeting different viral or host factors or working synergistically on the same pathway, can lower that risk from multiple directions. For SARS-CoV-2, pairing an M^pro^ inhibitor like nirmatrelvir with an RdRp inhibitor such as remdesivir not only gives synergy by hitting distinct replication steps, but also suppresses escape variants that would emerge under selective pressure from monotherapy [14]. Another elegant strategy exploits the virus’s own proofreading machinery. By using specific inhibitors to block the ExoN exonuclease and thereby disrupt the error correction system, nucleoside NAs become more effective against ExoN-mediated resistant mutants [15].

Successful combination therapy involves more than simply picking two active drugs. Their mechanisms must be truly orthogonal, otherwise resistance is only slowed. Pharmacokinetic compatibility is equally critical, as without sustained synergistic concentrations in the body, real synergy is hard to achieve. Preclinical synergy scores also need a clean safety margin. When these conditions come together, combination therapy becomes a powerful strategy, not just for SARS-CoV-2 but for any complex disease where resistance keeps evolving.

Four landmark studies have delineated two core SARS-CoV-2 resistance mechanisms: M^pro^ active-site mutations and ExoN-mediated RNA proofreading. Structure-guided design (exemplified by Jun13698) enables effective evasion of resistance mutations, while a mechanistic understanding of ExoN supports the rational optimization of NAs (e.g., bemnifosbuvir). These advances establish an integrated paradigm for antiviral development spanning mechanism dissection, target validation, rational drug design, and combination therapy. Future efforts should focus on improving druggability, optimizing combination regimens to mitigate resistance, monitoring the dynamics of clinical resistance, and developing broad-spectrum inhibitors targeting conserved regions of coronavirus. Meanwhile, the latest strategies and new technologies in the field of antiviral drugs [16], including artificial intelligence technology, as well as the latest understanding of drug resistance [17], should be promptly applied to prepare for the development of drugs against emerging coronaviruses and other newly emerging and acute viruses, including Nipah virus [18], Chikungunya virus, Hantavirus, etc., during the first half of 2025–2026.
